# Low frequency visual stimulation enhances slow wave activity without disrupting the sleep pattern in mice

**DOI:** 10.1038/s41598-022-16478-8

**Published:** 2022-07-19

**Authors:** Stephen Thankachan, Chun Yang, Ksenia V. Kastanenka, Brian J. Bacskai, Dmitry Gerashchenko

**Affiliations:** 1 Veterans Affairs Boston Healthcare System, Harvard Medical School, West Roxbury, MA 02132 USA; 2 Department of Neurology, MassGeneral Institute of Neurodegenerative Diseases, Massachusetts General Hospital, Harvard Medical School, Charlestown, MA 02129 USA

**Keywords:** Biological techniques, Optogenetics, Neuroscience

## Abstract

Non-invasive stimulation technologies are emerging as potential treatment options for a range of neurodegenerative disorders. Experimental evidence suggests that stimuli-evoked changes in slow brain rhythms may mitigate or even prevent neuropathological and behavioral impairments. Slow wave activity is prevalent during sleep and can be triggered non-invasively by sensory stimulation targeting the visual system or directly via activation of neurons locally using optogenetics. Here, we developed new tools for delivering visual stimulation using light-emitting diodes in freely moving mice while awake and during sleep. We compared these tools to traditional optogenetic approaches used for local stimulation of neurons in the cerebral cortex. We then used these tools to compare the effects of low-frequency visual versus optogenetic stimulations on the slow wave activity and sleep pattern in mice. Visual stimulation effectively enhanced slow wave activity without disrupting the sleep pattern. Optogenetic stimulation of cortical GABAergic neurons increased NREM sleep. These results suggest that visual stimulation can be effective at boosting slow wave activity without having adverse effects on sleep and thus holds great potential as a non-invasive stimulation treatment strategy.

## Introduction

The emergence of non-invasive stimulation modalities as therapeutic interventions holds promise for neurodegenerative disorders. Sleep-dependent brain rhythms, slow oscillations, are important for plasticity-related processes during sleep^1,2^. Slow oscillations are driven by cortico-thalamic circuit in the delta frequency range. Experimental evidence suggests that stimuli-evoked changes in brain dynamics at the slow oscillation frequency may slow down or even reverse some neuropathological and behavioral impairments. For example, stimulating the brain at a slow frequency (about 0.75 Hz or phase-locked to the slow oscillations) with transcranial direct current stimulation or auditory tones enhanced the slow oscillation power and improved memory performance in young and older adults^3,3–7^. Local stimulation of cells in the cerebral cortex at 0.6 Hz halted amyloid plaque deposition and prevented calcium overload associated with this pathology in the APP/PS1 animal model of Alzheimer’s disease (AD)^8^. Most previous studies employed either local stimulation of cortical circuits or auditory stimulation to enhance slow wave activity. Visual stimulation represents an alternative and convenient option for achieving sensory stimulation during sleep, however, it received little attention.

Here we developed LED-driven visual and optogenetic stimulation protocols. We compared their effects on EEG and sleep patterns in freely moving mice. Visual stimulation is often performed using a liquid–crystal display (LCD) placed in front of a mouse^9–11^, or using optical fibers placed distally from eyes of an animal and connected to a light-emitting diode (LED) driver that is controlled by a pulse generator^12^. These methods of visual stimulation generally involve restraining the animal. In some studies, visual stimulation has been performed in unrestrained mice by placing them in an arena surrounded by LED screens^13^ or a dark chamber illuminated by a LED bulb flickering at various frequencies^14,15^. In these studies, visual stimulation was performed in either awake or anesthetized mice.

Local optogenetic stimulation of neurons expressing light activated opsins in freely moving mice is usually performed by placing a fiber optic cannula into the desired brain area, which is subsequently connected to an optical fiber^16,17^. Optogenetic stimulation is then accomplished by the use of function generators controlling the drivers of the light source (LEDs or diode lasers) directly or by controlling an external electro-mechanical shutter in the path of the stimulation light beam. For neuronal activation, periodic pulses with a pulse width in the range of about 1–500 ms and frequency (repetition rate) in the range of 0.5–100 Hz are used. The typical diameter of a fiber optic cannula ranges from 200 to 400 µm, which allows illuminating a small area in the brain (less than 1 mm^3^).

The main goal of our study was to compare the effects of visual stimulation and local optogenetic stimulation of cortical neurons on EEG dynamics and sleep patterns. To achieve this goal, we developed two Arduino-based systems. One was used for performing visual stimulation and another was used for performing local stimulation of neurons in the cerebral cortex in freely moving mice. We tested these systems and assessed their advantages and limitations. The low frequency visual stimulation markedly enhanced slow wave activity without disrupting sleep. Local optogenetic stimulation of inhibitory GABAergic neurons in the cerebral cortex potentiated NREM sleep, while decreasing time spent awake.

## Results

### Design of the system for visual stimulation in freely moving mice

**Figure 1:**
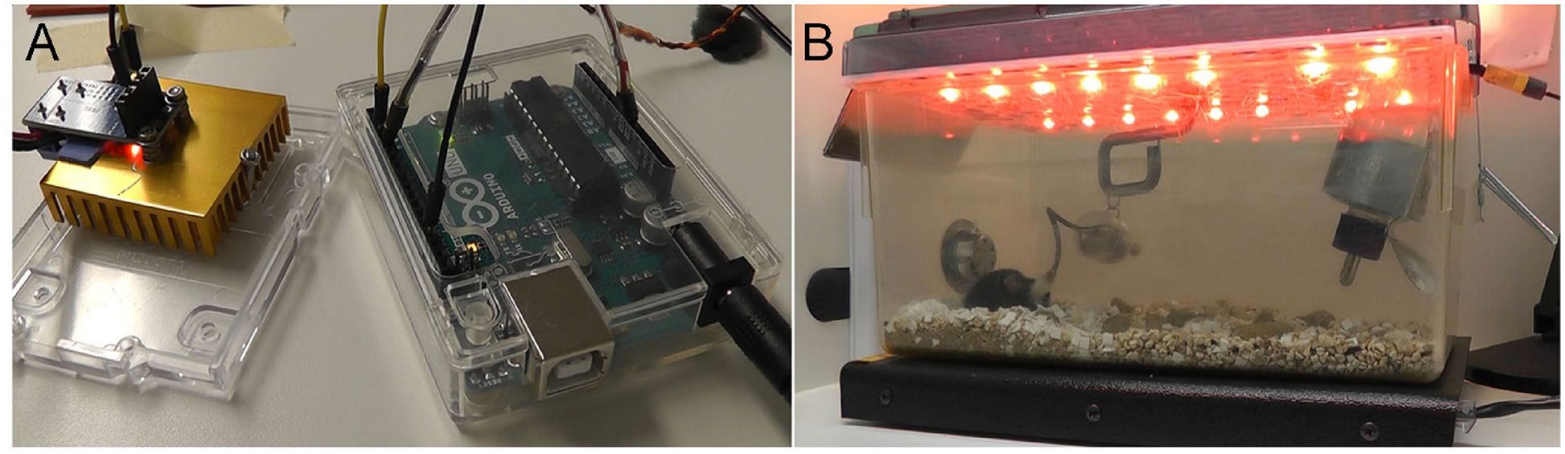
System for visual stimulation in freely moving mice. Arduino Uno and metal–oxide–semiconductor field-effect transistor (MOSFET) are used to generate square pulses of electric current (**A**) that power LED lamps mounted on the perforated aluminum plate at the top of a regular mouse cage (**B**).

The system for visual stimulation included four main components: Arduino Uno controller (catalog #A000066, Arduino; Fig. 1A), 15A 400 W MOSFET trigger switch module (Arduino; Fig. 1A), high power LEDs mounted on a perforated aluminum plate (Fig. 1B), and DC adjustable switching power supply (60 V, 5A, catalog #TP6005E, Tekpower). In this study, we performed visual stimulation using the following two types of high power LEDs: red (620 nm, 5 W, 1200 mA, DC 2–2.4 V, Chanzon) and far red (730 nm, 5 W, 1200 mA, DC 1.8–2.0 V, Chanzon). Supplementary materials contain programs used to trigger light pulses.

As an initial step towards optimizing light intensity for visual stimulation, we measured light intensity produced by our system. Then, we performed visual stimulation to excite cortical cells (via visual pathway) using 620-nm or 730-nm LED light source mounted on a perforated aluminum plate that was placed on the top of the mouse cage. Arduino UNO was used to generate pulses to drive the LED light stimulation. Power spectral density (PSD) was analyzed on the day when we performed visual stimulation. PSD was also analyzed at baseline prior to LED stimulation and the next day when LEDs were on.

### Measurements of light intensity for visual stimulation studies

620-nm and 730-nm light intensity was measured inside the cage using digital power and energy meter console (Si Sensor, 400–1100 nm, catalog #PM120D, Thorlabs) and a digital luxmeter (catalog #LX-1010B). The sensor was placed on the bottom of a standard mouse home cage, and a perforated aluminum plate with attached LEDs was positioned on the top of the cage. The light intensities measured in different locations of the cage are shown in Supplementary Fig. 1. The light intensity was similar in all locations of the cage, although it was somewhat higher in the center (up to 18 µW/mm^2^ or 10,000 Lux for the 620-nm light). Such high intensity of the red light was needed because mice have a low sensitivity to the 620 nm light^18^. The intensity of 730-nm light was higher (up to 140 µW/mm^2^ or 725 Lux) around the cage. 730-nm light served as control because this wavelength is outside of the visual light spectrum range for mouse vision^18^.

### Effect of visual stimulation on EEG power spectrum and sleep amounts

**Figure 2:**
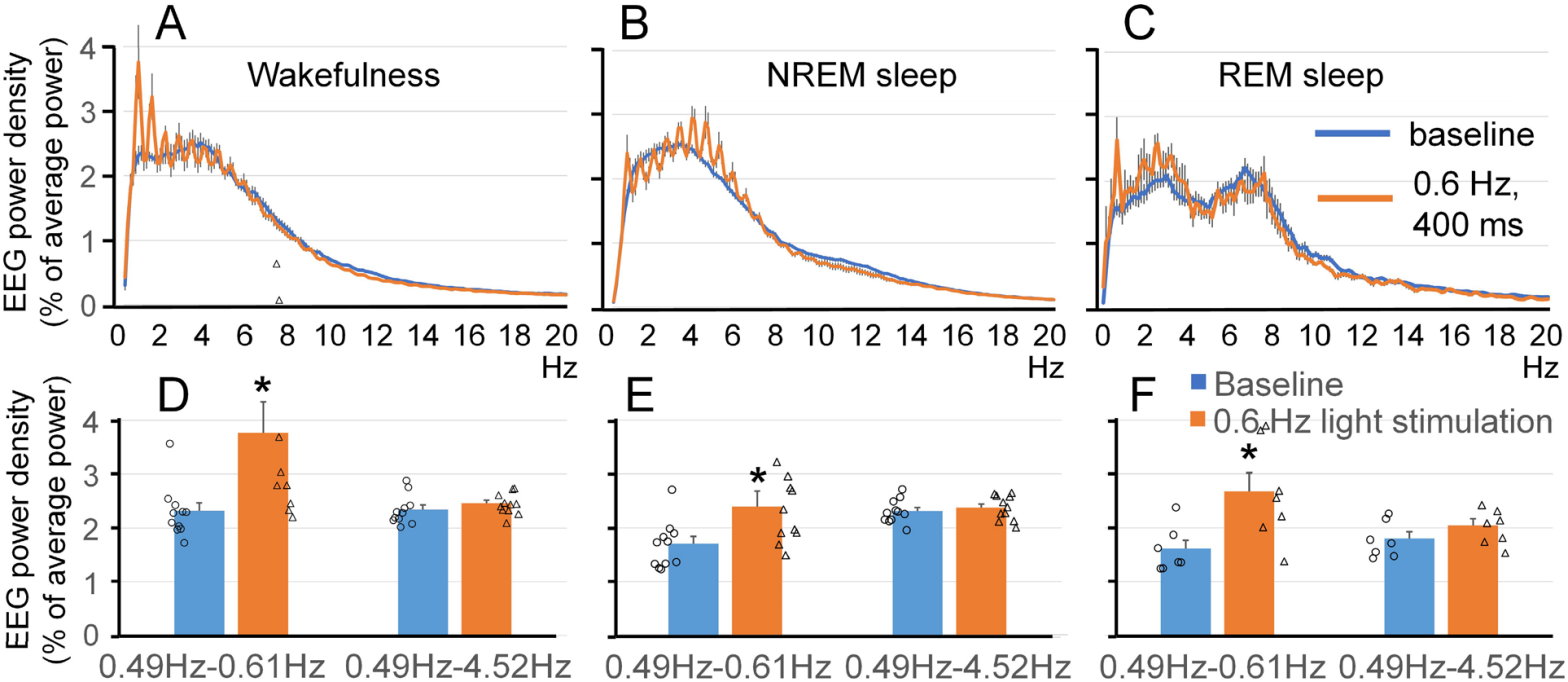
Effect of visual stimulation with 400-ms 620-nm light pulses at 0.6 Hz on EEG power spectrum. A peak at the fundamental frequency of 0.6 Hz and multiple harmonics as an integer of this frequency were present during visual stimulation. A similar response to visual stimulation was observed on the spectrogram during wakefulness (**A**), NREM sleep (**B**) and REM sleep (**C**). EEG power in the stimulation frequency range (0.49–0.61 Hz) was higher during the light stimulation than baseline in all three states, wakefulness (**D**), NREM sleep (**E**) and REM sleep (**F**), whereas EEG power in the delta frequency range (0.49–4.52 Hz) did not change significantly. *p < 0.05.

**Figure 3:**
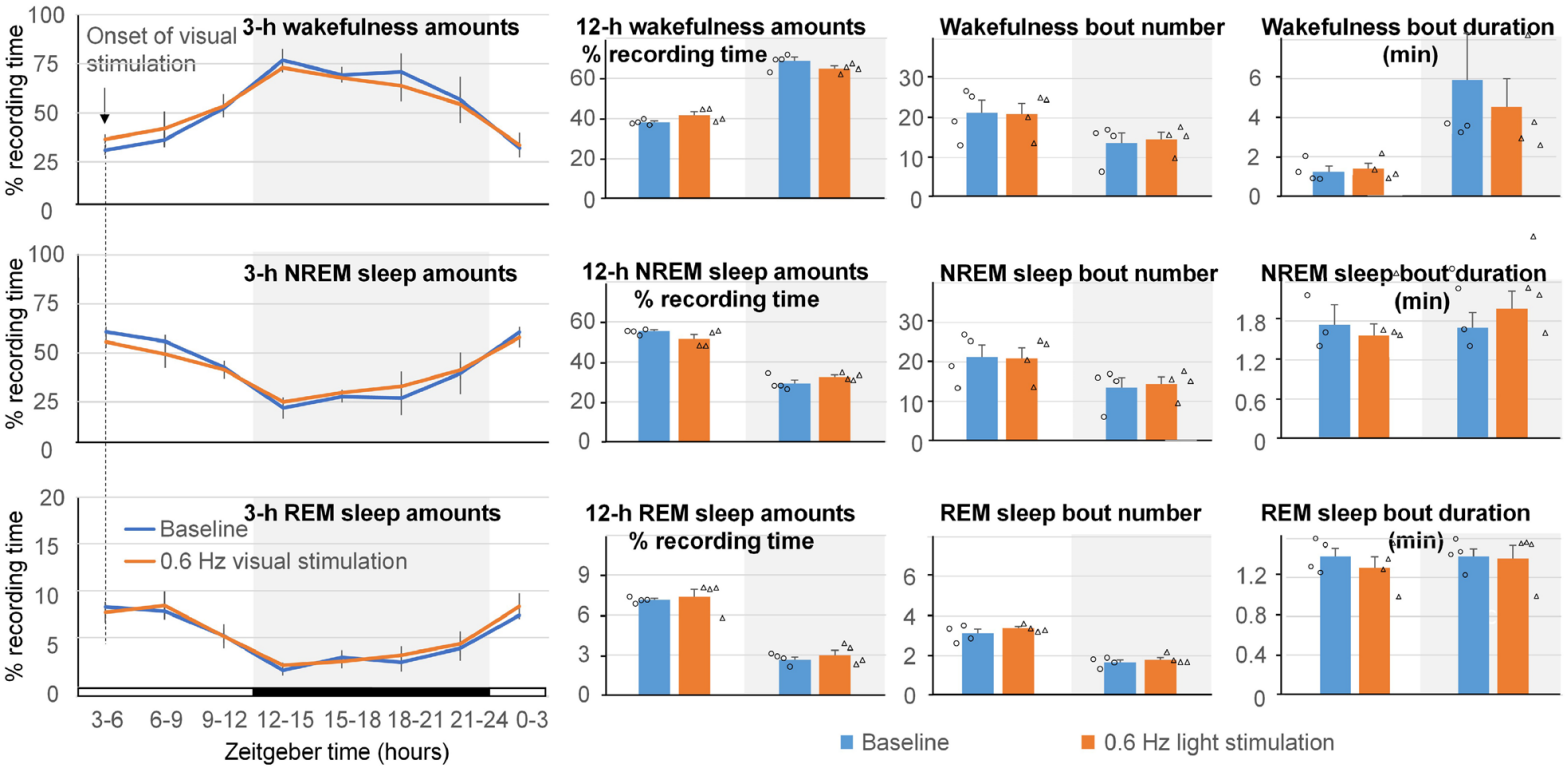
Effect of visual stimulation with 400-ms 620-nm light pulses at 0.6 Hz on sleep pattern. No significant differences in the amounts of wakefulness, NREM sleep and REM sleep calculated in either 3-h or 12-h intervals between the baseline and stimulation day were found. Number and duration of sleep bouts also did not change. White background indicates light periods, and grey background indicates dark periods.

**Figure 4:**
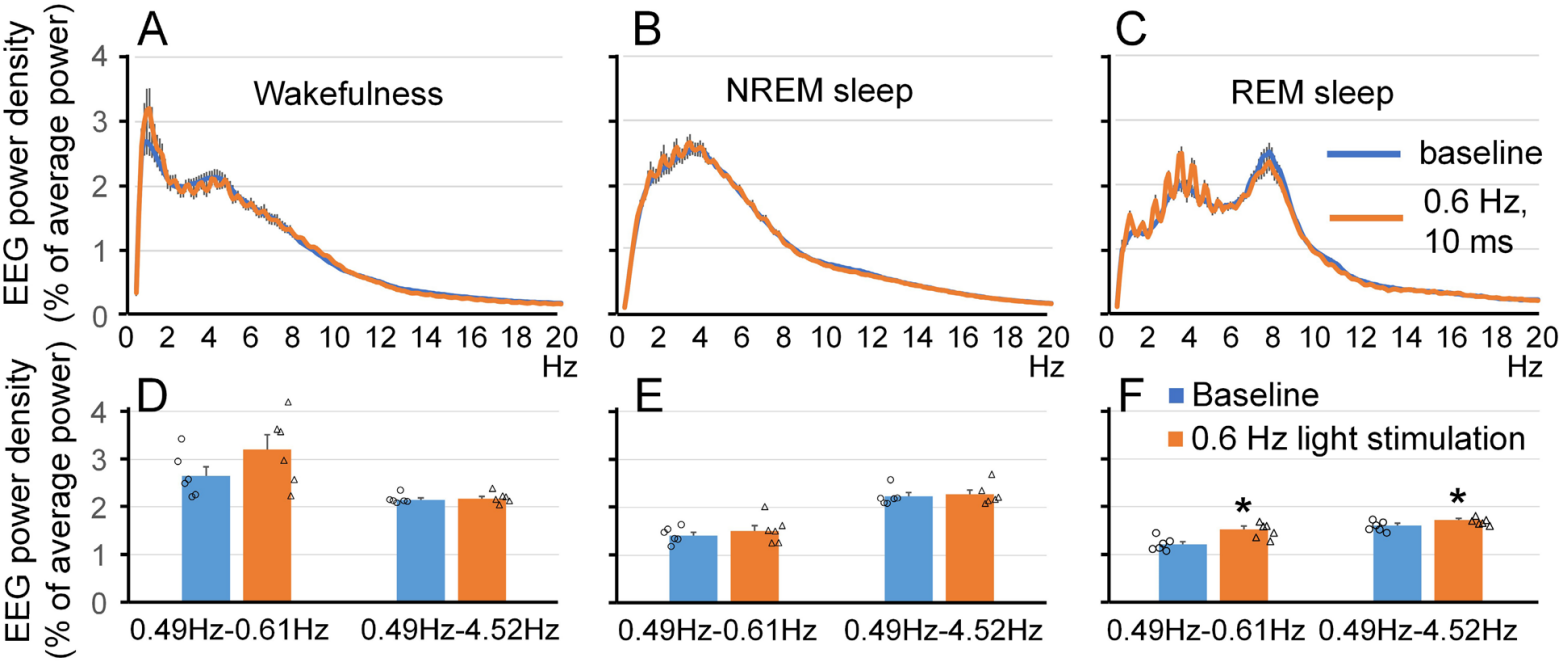
Effect of visual stimulation with 10-ms 620-nm light pulses at 0.6 Hz on EEG power spectrum. The profile of spectral power over 24 h was compared between the baseline day and the next day during which the visual stimulation was performed. The profile of spectral power in mice stimulated with 400-ms light pulses during wakefulness (**A**), NREM sleep (**B**) and REM sleep (**C**) is similar to that observed in the mice stimulated with 400-ms light pulses (Fig. 2), except that the amplitude of both the fundamental frequency and harmonics is lower. EEG power in the stimulation frequency range (0.49–0.61 Hz) and delta frequency range (0.49–4.52 Hz) were not statistically different between baseline and in the process of the light stimulation during wakefulness (**D**) and NREM sleep (**E**) but was higher during REM sleep (**F**). *p < 0.05.

**Figure 5:**
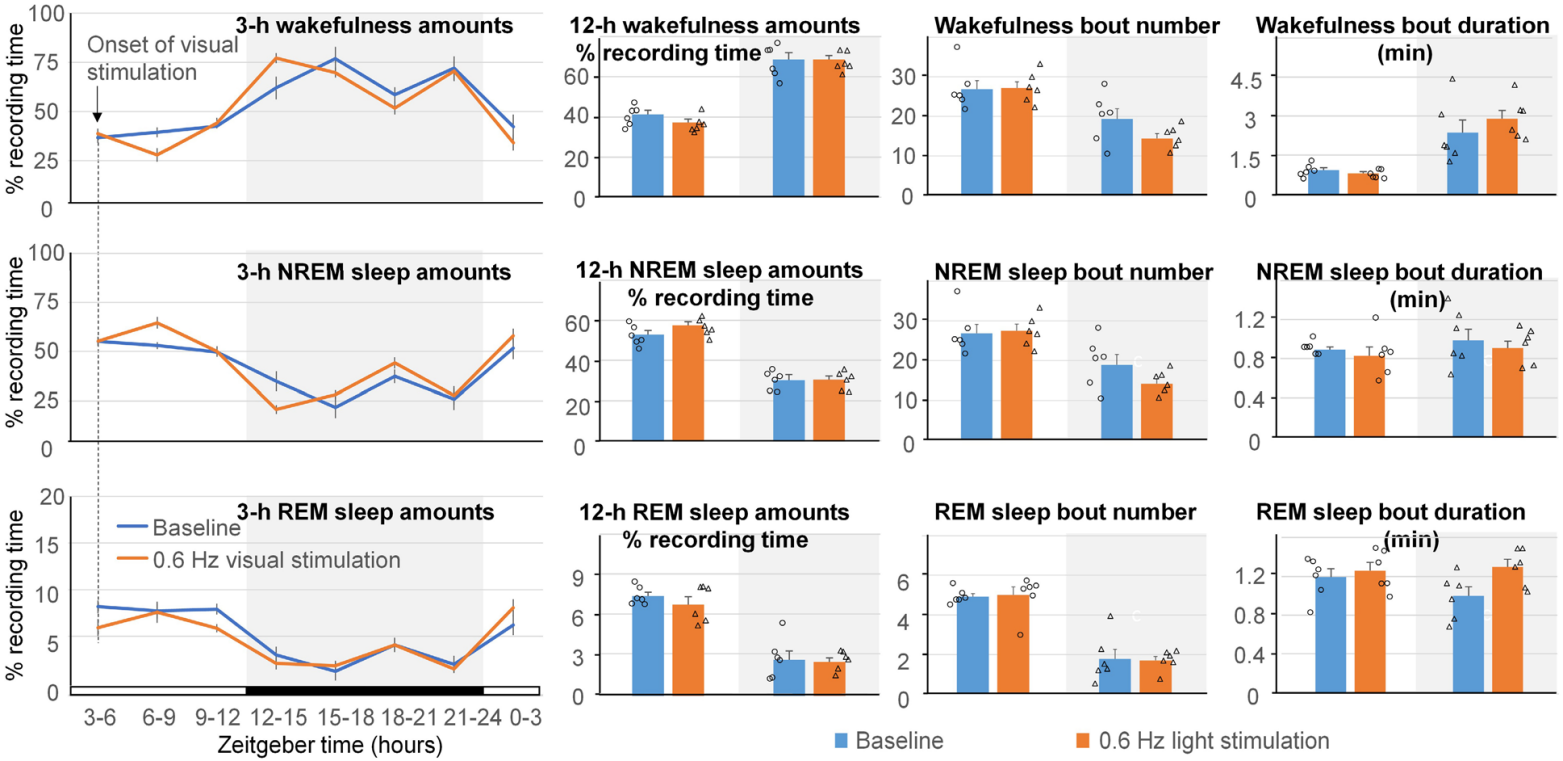
Effect of visual stimulation with 10-ms 620-nm light pulses at 0.6 Hz on sleep pattern. No significant differences in the amounts of wakefulness, NREM sleep and REM sleep calculated in either 3-h or 12-h intervals between the baseline and stimulation day were found. Number and duration of sleep bouts also did not change. White background indicates light periods, and grey background indicates dark periods.

We tested whether visual stimulation with LEDs can be effective at increasing power of slow oscillations without altering sleep dynamics. Visual stimulation with 400-ms light pulses of 620-nm light at the frequency of 0.6 Hz produced a peak at the stimulation frequency at 0.6 Hz, the endogenous frequency of slow oscillations, and multiple harmonics (Fig. 2A,B,C). The power in the stimulation frequency range induced by the 400-ms light pulses was significantly higher during the light stimulation compared to baseline (Fig. 2D,E,F) in all the three states; wakefulness (64.4 ± 24.6% increase, t(10) = − 2.595, *p* = 0.027), NREM sleep (45.6 ± 21.4% increase, t(10) =  − 2.280, *p* = 0.046), and REM sleep (72.3 ± 27.7% increase, t(6) =  − 2.842, *p* = 0.029). Significant power differences were also observed at faster frequencies. Theta power was higher during NREM sleep in mice stimulated with 400-ms 620-nm light pulses at the frequency of 0.6 Hz (Fig. 2B; 5.5 ± 1.9% increase, t(10) =  − 2.727, *p* = 0.021). No significant differences in the amounts of wakefulness, NREM sleep and REM sleep, calculated in 3-h intervals between the baseline and stimulation day were found (Fig. 3). When visual stimulation was performed with shorter light pulses (10-ms) of 620-nm light at the frequency of 0.6 Hz, we observed changes in the EEG power spectrum that were similar to those produced by the 400-ms light pulses, such as the peak at stimulating frequency of 0.6 Hz and multiple harmonics were clearly visible during wakefulness, NREM sleep and REM sleep (Fig. 4A,B,C). However, the power of both the 0.6 Hz peak and harmonics was lower when the duration of light pulses was shorter. When EEG power in the stimulation frequency range (0.49–0.61 Hz) and delta frequency range (0.49–4.52 Hz) was compared between baseline (i.e., one day prior to the light stimulation) and during the light stimulation (Fig. 4D,E,F), significant differences were observed during REM sleep (stimulation frequency, 26.0 ± 4.0% increase, t(5) =  − 7.067, *p* = 0.001; delta frequency, 7.2 ± 1.5% increase, t(5) =  − 5.115, *p* = 0.004). No significant differences in sleep amounts, or bout numbers or bout durations were found (Fig. 5). Visual stimulation with 400-ms 620-nm light pulses at the frequency of 1.2 Hz also produced changes in the EEG power spectrum (Supplementary Fig. 2A,B,C), whereas the light outside the mouse visual spectrum (730 nm) did not affect the EEG pattern (Supplementary Fig. 2D,E,F). Thus, visual stimulation at the endogenous frequency of slow oscillations was effective at increasing the power of slow waves without disrupting the sleep pattern in mice.

### Design of optogenetic system for activating neurons locally in the cerebral cortex

**Figure 6:**
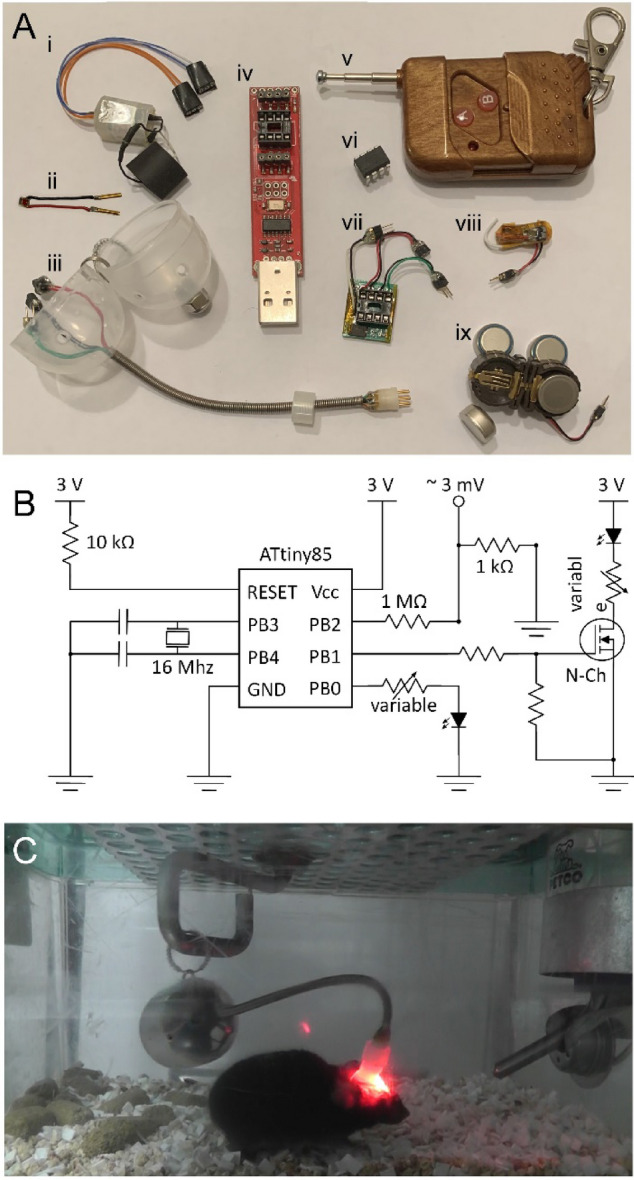
Optogenetic system for activating neurons locally in the cerebral cortex in mice. Components of the system are shown in A. Biopotentials are recorded by the transmitter (i). Light stimulation is produced by a high-power LED (ii). The system employs a special apparatus for connecting with the animal (iii). A programmer is used to generate pattern of optogenetic stimulation (iv). A remote controller is used to turn the optogenetic system on or off (v). ATTINY85 microcontroller (vi), PCB supporting ATTINY85 socket, crystal oscillator, and resistors for controlling the output voltage (vii), 433 MHz receiver module (viii), and battery holder with 675 zinc-air batteries (ix) are required to operate the system. A circuit diagram for generating different patterns of signals is presented in B. Pins PB3 and PB4 of the ATTINY85 microcontroller are used for connecting with the external crystal oscillator. Pins PB0, PB1 and PB3 can be programmed to power an LED either directly (PB0) or via a N-channel MOSFET (PB1). Additionally, a low voltage signal can be generated (PB2). Simultaneous recording of EEG/EMG and optogenetic stimulation in a freely moving mouse were performed in a regular mouse cage placed on the top of a wireless receiver (C). ATTINY85-based circuit and batteries were located in a container connected with the mouse. It was supported by the apparatus that was counterbalanced and rotated circularly to eliminate pressure on the animal.

We next set out to compare the effect of visual stimulation with that of optogenetic stimulation targeted to inhibitory cortical neurons. Slow oscillations make transitions between bursts of vigorous activity, termed UP states, and periods of relative quiescence, termed DOWN states. Inhibitory neurons temporary group or synchronize neuronal activity and are important in producing reliable network transitions to DOWN states^19,20^. Thus, inhibitory interneurons were targeted optogenetically to potentiate slow wave activity. Figure 6A shows the components of optogenetic system for activating neurons in the cerebral cortex. Contrary to other commonly used optogenetic systems, it does not require function generators, LED drivers, lasers, fiber-pigtailed LEDs, fiber optic patch cables and cords, and fiber optic rotary joints. Instead, most components of the system are commercially available on the market for purchase and are inexpensive. The system consists of the following: a biopotential transmitter (PhysioTel F20-EET, Data Sciences International (DSI); Fig. 6A-i), a high-power LED (629 nm, catalog #L1C1-RED1000000000, Lumileds; Fig. 6A-ii), an apparatus for connecting to freely moving mice (Neurotargeting Systems, Fig. 6A-iii), a tiny AVR Programmer (catalog #PGM-11801, SparkFun Electronics; Fig. 6A-iv), a 433 MHz RF wireless remote controller (wireless remote control switch transmitter and receiver kit, Anntem; Fig. 6A-v), a ATTINY85 microcontroller (catalog #ATTINY85-20PU, Microchip Technology; Fig. 6A-vi), a printed circuit board (PCB) supporting ATTINY85 socket, a crystal oscillator, resistors for controlling the output voltage (Fig. 6A-vii), 433 MHz receiver module (wireless remote control switch transmitter and receiver kit, Anntem; Fig. 6A-viii), and a battery holder with four 675 zinc-air batteries (catalog #BC501SM-TR, Memory Protection Devices; Fig. 6A-ix).

Figure 6B shows a circuit diagram of the described system. Periodic light pulses were generated using the ATTINY85 microcontroller (catalog #ATTINY85-20PU, Microchip Technology). This microcontroller is able to generate high-quality signals. We generated different signal patterns and calibrated them using an oscilloscope. Resistors were used to reduce voltage of the signals. ATTINY85 has an internal oscillator which runs at 8 MHz (or 16 MHz using internal phase locked loop). The internal oscillator works well for performing most research tasks, but it is not sufficiently accurate for applications that require precise temporal control (e.g., microsecond precision). To allow such applications, we clocked ATTINY85 with the external crystal oscillator. When we compared the time determined by the external crystal oscillator with the precise internet time using https://time.is/, the synchronization was highly accurate. We observed the discrepancy of less than a second within the 24-h period.

Figure 6C depicts the system using the PhysioTel F20-EET telemetry transmitter designed for small animals such as mice (DSI). Alternative types of transmitters and loggers could be used in this system, depending on their availability in each particular laboratory and goals of the study.

### Light intensity and temperature increases produced locally by the 629-nm LED

Whereas light delivered via an optical fiber generates small amounts of heat due to light absorption^21^, an LED can generate additional amounts of heat within the LED device itself due to the inefficiency of the semiconductor processes that generate light. Therefore, we measured both light intensities produced by the 629-nm LED and corresponding increases in temperature. Head implants with 629-nm LEDs (catalog #L1C1-RED1000000000, Lumileds) powered by zinc-air batteries (675P, ZeniPower) or CR2 lithium manganese dioxide battery (EL1CR2BP, Energizer) were used as the light power source for measuring light intensity and temperature. Energy density and specific energy per weight is higher in zinc-air batteries than lithium-based batteries, but lithium-based batteries can deliver much higher current than zinc-air batteries. Therefore, we tested both types of batteries. Digital power and energy meter console (Si Sensor, 400–1100 nm, catalog #PM120D, Thorlabs) was used to measure the light power, whereas a temperature probe (Digital Multimeter MM600, Klein Tools) right below the LED was used for accessing the temperature changes (Supplementary Fig. 3).

Supplementary Fig. 4 shows changes in the temperature during various intensities of the LED light. The power of LED light was adjusted by selecting the appropriate resistor (2.2, 3.9, 8.2 or 22.0 Ω). When activated at frequency of 0.6 Hz, LEDs generated the temperature increases that were below the detection limit for the 10-ms light pulses (Supplementary Fig. 4A) and less than 1.5 °C for the 400-ms light pulses (Supplementary Fig. 4B). Temperature increases were directly proportional to the duration of the LED powered on (Supplementary Fig. 4C) and light intensity (Supplementary Fig. 4D).

We also measured attenuation of light intensity by placing a piece of mouse skull or 1-mm slice of mouse brain between the LED and digital power & energy console. Mouse skull reduced 629-nm light intensity by 24 ± 2%, and 1-mm brain slice reduced light intensity by 58 ± 3%. Based on the obtained results, we selected the parameters of LED activation that were sufficient to activate neurons locally while avoiding undesirable effects of overheating the brain tissue, and thus used light with 65 mW/0.6 Hz/10 ms (experiment 3) and 28 mW/0.6 Hz/400 ms (experiment 4).

### Effect of local activation of GABAergic neurons in the cerebral cortex on EEG power spectrum and sleep amounts

**Figure 7:**
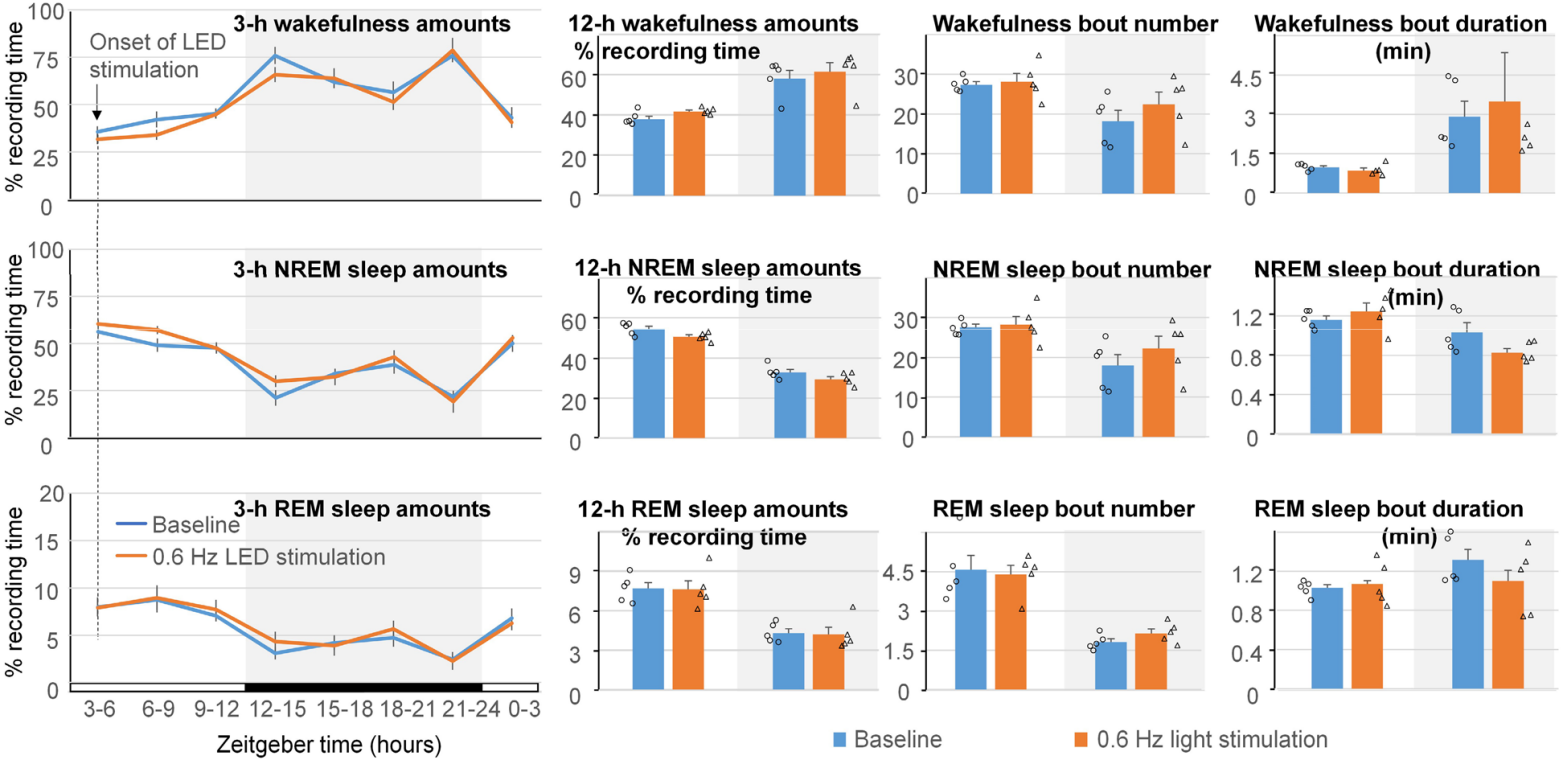
Effect of 24-h local stimulation of ChrimsonR containing neurons in the cerebral cortex with 400-ms 629-nm light pulses at 0.6 Hz on sleep pattern. No significant differences in the amounts of wakefulness, NREM sleep and REM sleep calculated in 3-h intervals between the baseline and stimulation day were found. Number and duration of sleep bouts also did not change significantly. However, an increase in the amounts of NREM sleep and reduction in the amounts of wakefulness were observed during the 12-h light on period. White background indicates light periods, and grey background indicates dark periods. *p < 0.05.

**Figure 8:**
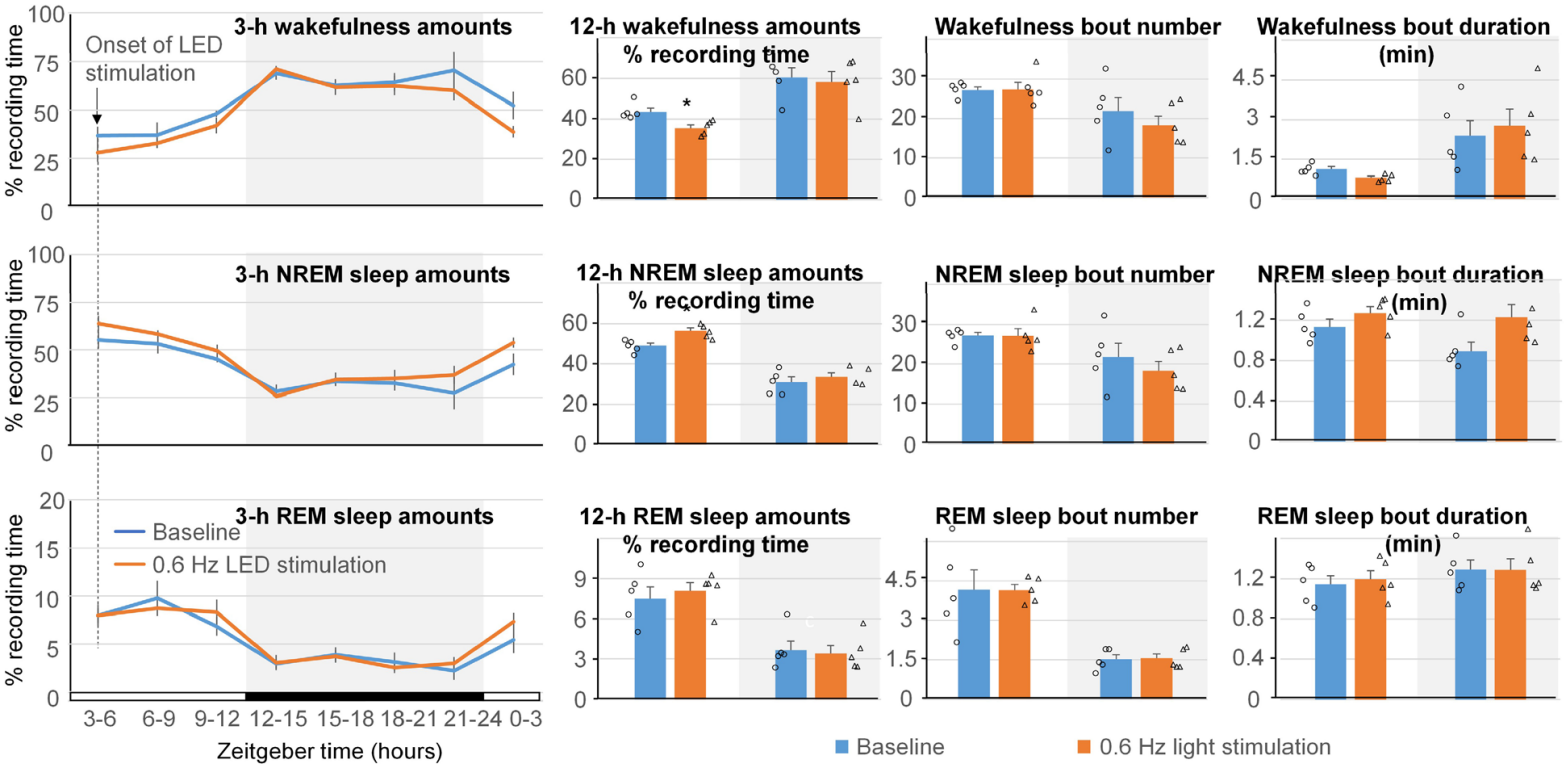
Effect of local stimulation of ChrimsonR containing neurons in the cerebral cortex with 10-ms 629-nm light pulses at 0.6 Hz on sleep pattern. No significant differences in the amounts of wakefulness, NREM sleep and REM sleep calculated in either 3-h or 12-h intervals between the baseline and stimulation day were found. Number and duration of sleep bouts also did not change. White background indicates light periods, and grey background indicates dark periods.

Viral delivery of AAV2/5-Syn-FLEX-rc[ChrimsonR-tdTomato] was targeted into the cerebral cortex at the depth of 0.8 mm, so the virus was concentrated in the deep layers of the cerebral cortex and also spread into the hippocampus (Supplementary Fig. 5). Local stimulation of cortical ChrimsonR-containing neurons with 400-ms light pulses at the frequency of 0.6 Hz in the mice injected with the AAV produced a small increase in the amounts of NREM sleep (7.5 ± 1.6%; *t*(4) = − 4.191, *p* = 0.014) and a reduction in the amounts of wakefulness (− 8.1 ± 1.9%; *t*(4) = 4.656, *p* = 0.010) during the light on period (Fig. 7). Sleep amounts during the light off period, and also bout numbers and bout durations, were not significantly different from those recorded during the preceding baseline day. Shorter pulses of 620-nm light (10 ms) at the same frequency of 0.6 Hz did not have an effect on sleep amounts, bout numbers or bout durations (Fig. 8).

Changes of the EEG power were also observed in the mice in which GABAergic neurons were stimulated locally with 400-ms light pulses at the 0.6 Hz frequency. In these mice, the peak at 0.6 Hz and multiple harmonics were present on the spectrogram (Supplementary Fig. 6, orange line). Because LEDs on the top of the skull had to be powered at about 2.8–3.0 V, electrical interference could occur with EEG potentials that have several orders lower voltage (i.e., millivolt range). To test such a possibility, we implanted three C57BL/6 J male adult mice with the 629-nm LED, EEG and EMG electrodes. The implantation procedure was identical to that used in the experiment 3, except that we did not inject the mice with the AAV. Thus, these mice did not have ChrimsonR-expressing neurons in the cerebral cortex that could respond to the pulses of red light. Nevertheless, both fundamental and harmonic components on FFT-generated power spectrum were present in these mice (Supplementary Fig. 6, blue line). Thus, optogenetic stimulation was effective at increasing the power of slow oscillations, while potentiating NREM sleep in mice.

### Effect of local activation of GABAergic neurons in the cerebral cortex on expression of cFos

To demonstrate that activation of GABAergic neurons in the cerebral cortex can be achieved using LEDs placed on the top of the skull, we injected C57BL/6 J mice with AAVs expressing either ChrimsonR-RFP (n = 3) or RFP (n = 3), exposed them to 400-ms 629-nm light pulses at 0.6 Hz for 2–2.5 h, perfused them with formalin, and used brain sections for cFos/RFP double staining. We observed a high induction of cFos expression in mice expressing ChrimsonR-RFP but not control mice expressing only RFP (Supplementary Fig. 7).

### FFT analysis of non-sinusoidal waveforms: harmonic generation

To demonstrate that harmonics are expected in the EEG power spectrum when non-sinusoidal periodic stimulus are used, we performed FFT analysis of artificially produced sine waves and square waves using SleepSign software (Kissei Comtec). Sine waves were generated by a signal generator (catalog # 890,625, Warner Instruments, Hamden, CT), and square waves were generated by the ATTINY85 microcontroller. The signals were recorded using the same F20-EET wireless transmitters (DSI) that were used to record EEG and EMG in present studies. As expected, FFT analysis of sine waves resulted in one peak at the stimulation frequency (Supplementary Figs. 8, 9, 10, 11). When square waves were analyzed, we observed both fundamental and harmonic components on the power spectrum (Supplementary Figs. 8, 9, 10, 11). The result was similar when the analysis was performed using different software (multi-taper spectral analysis script, MatLab and IGOR Pro software, WaveMetrics) (Supplementary Figs. 12, 13).

## Discussion

A better understanding of the effect of different oscillatory patterns on brain functioning is needed for development of new efficient therapies for neurodegenerative disorders. Recent studies demonstrated that exposing Alzheimer’s mouse models to LED lights flickering at 40 Hz stimulated gamma waves and reduced levels of beta-amyloid and tau proteins^14^. While enhancing gamma oscillatory activity may be beneficial during wakefulness, experimental evidence suggests that enhancing a slow oscillatory activity should be beneficial during sleep^22,23^. Slow waves in the cerebral cortex can be triggered either directly by activation of neurons locally in the brain by optogenetic stimulation, transcranial magnetic stimulation, transcranial direct current stimulation, or indirectly by sensory stimulation (auditory, tactile, vestibular) during sleep^8,23–25^. Regardless of the way by which slow waves are triggered, it is thought that they originate locally in distinct cortical areas and then spread from the site of origin throughout the cerebral cortex as travelling waves^26,27^. Efficient tools for changing oscillatory dynamics in the brain of freely behaving mice via sensory stimulation are needed for testing neurobehavioral effects of slow wave induction. Visual stimulation holds a particularly great promise as an efficient way of sensory stimulation because it can be easily delivered during sleep. In the present study, we produced systems for either visual stimulation or local stimulation of neurons in the brain and compared their efficiency in changing brain oscillatory pattern and sleep in mice.

Although white light was used in previous studies to perform visual stimulation^14,28^, we used red light LEDs (620 nm) in our system. Red light penetrates better through the tissue than light with a shorter wavelength, so it could be used to stimulate the mouse with closed eyelids during sleep. We delivered light at the intensity that was higher than in previous studies (up to 18 µW/mm^2^, 10,000 Lux) because mouse’s sensitivity to 620-nm light is lower than sensitivity to light with shorter wavelengths^18^. When mice were exposed to both 400 ms and 10 ms pulses of light at the frequency of 0.6 Hz, clear changes in the EEG power spectrum were present (Figs. 2 and 4). However, longer pulses of red light (400 ms) were more efficient than shorter pulses (10 ms) in producing slow waves, as we observed an increase of power in the 0.6-Hz frequency bin during wakefulness, NREM sleep and REM sleep (Fig. 2). A likely explanation of this finding is that more neurons are activated during long pulses of light. This results in generation of traveling slow waves of a higher amplitude because more neurons are recruited in a feed forward way in this oscillatory pattern.

Visual stimulation did not disrupt the sleep pattern in the mice (Figs. 3 and 5). This is inconsistent with other studies^29,30^ and could be due to different experimental conditions of our study (pulses of red light delivered at low rate). Previous studies suggested differential role of blue and green light in the regulation of sleep. Blue light (470 nm) caused behavioral arousal, whereas green light (530 nm) produced rapid sleep induction^31^. Although both green and blue light elevated levels of the stress hormone corticosterone in the blood of mice exposed to the light compared with mice kept in the dark, corticosterone levels in response to blue light were higher than levels in mice exposed to green light^31,32^. Our understanding of the role of red light in sleep modulation remains limited and requires additional studies.

Visual stimulation at 0.6 Hz produced an increase in the stimulus frequency F. It also elicited multiple harmonics at an exact integer multiple of the stimulus frequency, such as 2*f*, 3*f*, and so on (Figs. 2 and 4). This result can be explained by production of evoked potentials in the visual cortex leading to occurrence of fundamental and harmonic components on FFT-generated power spectrum that were described in both rodents and humans^33–36^. When the time waveform is periodic but not sinusoidal, the response spectrum will contain narrow peaks at exact integer multiples of the input frequency^34^. Due to the high importance of correct interpretation of the FFT analysis of non-sinusoidal signals, we decided to generate such signals using the ATTINY85-based electrical circuit and signal generator and recorded them using the DSI transmitter. When the signal was a perfect sine wave, we did not observe any higher harmonics in the power spectrum. In contrast, the FFT analysis of 10-ms and 400-ms square waves generated at several frequencies resulted in various patterns of higher harmonic responses (Supplementary Figs. 8, 9, 10, 11, 12, 13). Harmonic components of periodic non-sinusoidal signals could lead to power increase in a higher frequency range or coupling between lower and higher frequencies^37–39^. In our experiment, visual stimulation with 400-ms 620-nm light pulses at 0.6 Hz produced an increase in theta power (4–9 Hz) during NREM sleep. Because this increase was associated with the presence of harmonics, we believe that it should be interpreted as a consequence of FFT analysis of the 0.6-Hz of non-sinusoidal signal rather than a change in the biological processes that oscillate in theta frequencies.

As an alternative strategy, we targeted inhibitory cortical interneurons locally. We used an LED instead of a fiber optic cable with optical cannula. The new design allowed us reducing weight of the head implant and simplified experimental procedures. The LED fastened on the top of the skull weighed less than 20 mg, which is less than a typical weight of an optical cannula. Moreover, optical cannula allows illuminating a much smaller area in the cerebral cortex than an LED. We used an LED covering the area of 1 mm^2^, which is still much larger than the area of a typical optical cannula (from 0.002 to 0.3 mm^2^). It should be noted that our system allows placing several LEDs, which could be used to stimulate large areas in the cerebral cortex. The LED was connected by thin wires to zinc-air batteries and a microprocessor for controlling the light flashing pattern. Using this design, we achieved the light power density in the brain of more than 5–10 mW per mm^2^, which is typically used in optogenetic experiments. Temperature increase under the LED was negligible when 10-ms light pulses were delivered at the rate of 0.6 Hz (Supplementary Fig. 4A,C). The light stimulation patterns of 10 ms/20 Hz or 400 ms/0.6 Hz resulted in the local temperature increases on the top of the skull less than 1.5 °C (Supplementary Fig. 4B,D). Taken into account that temperature in the cerebral cortex would be affected even less than on the top of the skull, such small rise in temperature is unlikely to have a strong impact on neuronal activity in most of the studies. If longer pulses of light are used at a higher rate, caution should be taken not to overheat the brain tissue^40^. One more limitation of the use of LED in the optogenetic studies in mice is a potential interference in the EEG recordings. We observed artifacts on the EEG during the LED on/off transitions (Supplementary Fig. 6), but we expect that they could be greatly reduced by shielding the wires that are used for powering the LED.

It should be noted that some red light used for local optogenetic stimulation of neurons in the cerebral cortex could reach the animal’s eyes in our experiment (Video 1), raising the possibility that the effect of local stimulation on sleep was caused by visual stimulation. However, local optogenetic stimulation resulted in the increase of NREM sleep amounts (Fig. 7), whereas visual stimulation at a much higher intensity by the LEDs located at the top of the cage did not change sleep amounts (Fig. 3). This result suggests that the increase in NREM sleep was related to the local stimulation of neurons rather than to the indirect effect of visual stimulation in this experiment.

The mechanisms underlying the changes in the power spectrum and NREM sleep amounts observed in the present study may be related to the generation of traveling waves. Visual or optogenetic stimulation evokes a radial wave of activity in the cerebral cortex, which may be mediated by volleys of synaptic potentials that modulate excitability of individual neurons^41,42^ The wave characteristics could be affected by the factors that have an effect on the neuron excitability. The amplitude of visual evoked potentials greatly depended on whether visual stimulation was delivered during the day, night, or different times of day^43^. Cortical processing of sensory information by visual evoked responses were also found to vary as a function of the vigilance state, being of higher magnitude during slow wave sleep than wakefulness and REM sleep^44^. In addition, neuronal responses to different frequencies may be also different. The sleeping brain is unable to effectively synchronize large neuronal populations in response to rapid sensory stimulation (8 or 10 Hz), but can still respond to slow stimulation (3 or 5 Hz)^45^. Taken together, these observations may explain why 0.6 Hz stimulation increased NREM sleep amounts during the light on period when mice mostly sleep, but not during the light off period (Fig. 7). Another possible explanation of the changes in sleep pattern observed in this study may be the direct influence of cortical neurons on subcortical brain regions involved in sleep–wake control. A recent study demonstrated that a cortex-wide reduction in the output from layer 5 pyramidal neurons resulted in a markedly increased wakefulness and reduced rebound of EEG slow-wave activity after sleep deprivation^46^. Layer 5 pyramidal neurons have a wide range of efferent connections to target structures involved in sleep–wake control, thus providing a potential explanation of this result^46^.

Our presented systems are applicable for studies in animal models of neurodegenerative diseases. For example, APP/PS1 mice show an overall trend towards reduced power in the lower frequencies (delta and theta) and higher power in the faster frequencies (alpha and beta)^47^. In these mice, optogenetic stimulation of cortical neurons at the frequency of 0.6 Hz (400-ms pulse duration) slowed Alzheimer’s progression by reducing amyloid deposition and preventing neuronal calcium elevations in our earlier study^8^. Alternatively, optogenetic stimulation at 1.2 Hz (400-ms pulse duration) facilitated disease progression in the mouse model^48^. In the present study, no significant changes in the amounts of sleep were observed when LED light stimulation of GABAergic neurons in the cerebral cortex was performed with 10-ms light pulses at the frequency of 0.6 Hz (Fig. 8). However, LED light stimulation with a longer light pulses (400 ms) produced significant increases in the amounts of NREM sleep, while decreasing time spent awake, during the light on period (Fig. 7). Additional studies will be needed to assess the role of potentiating slow wave activity via visual or optogenetic stimulations in mouse models of Alzheimer’s disease. Of particular interest will be its effects on sleep deficits and the neuropathophysiology associated with the disease progression^8^. We anticipate that the presented new tools will be useful in achieving these goals.

## Materials and methods

### Animals

GAD2-cre mice (n = 11, C57BL/6 J background, stock #028,867) and C57BL/6 J mice (n = 8, stock #000,664) were obtained from Jackson Laboratories and bred in house. Mice were housed at 70–74°F and a 12-h light/dark cycle (7:00 AM–7:00 PM) with food and water ad libitum. All procedures were in accordance with VA & National Institutes of Health guidelines and were approved by the VA Boston Healthcare System Institutional Animal Care and Use Committee. Adherence to transparent reporting was followed through the updated Animal Research: Reporting of In Vivo Experiments (ARRIVE 2.0) guidelines.

### Stereotaxic surgery and viral injection

Mice were deeply anesthetized with isoflurane (1–3%) and fixed to a stereotaxic frame. Skin over the skull was incised and retracted to expose skull. Hole was drilled on the skull for viral injection into the cerebral cortex. Electroencephalogram (EEG) electrode was screwed into the skull above frontal cortex (AP = 1.7; ML =  ± 1), and a reference electrode was screwed into the skull above the cerebellum. Electromyography (EMG) electrodes were placed in the nuchal muscle. The electrodes were then connected to a plug (PlasticsOne) and fixed to the skull using dental cement. Stereotaxic injections of viral vector AAV2/5-Syn-FLEX-rc[ChrimsonR-tdTomato] (8.5 × 10^12^ GC/ml, Addgene) were performed using a 1-μl Hamilton syringe (7000 series; Hamilton) connected to 30-gauge tubing filled with mineral oil connected to 35G Blunt NanoFil needle (catalog no. NF35BL-2; WPI) for targeted injection of 0.5 μl into the unilateral cortex [AP = 1.0, medial–lateral (ML) = 2.0, ventral (V) = 0.8]. A 1-μl virus aliquot was backloaded via the tip of the injector needle, and the syringe was placed in a stereotaxic horizontal holder. The injector needle was inserted through the dura, placed above the target cortical region, and 0.5 μl of virus was slowly injected over 10 min. The injector needle was left in place for an additional 10 min to allow virus diffusion into the brain and to avoid backflow along the cannula/needle tract. Once viral injection was complete, the injector needle was removed, and a red-light LED (629 nm; Lumileds) was fastened on the top of the skull with clear acrylic cement above the cerebral cortex injected with the AAV. Following the injection and mounting of the electrodes and LED on the skull with dental cement, the scalp incision was sutured closed. The mice were injected with meloxicam and allowed to recover.

### In vivo EEG/EMG recordings

Mice were tethered to the F20-EET wireless transmitter (DSI) on a swivel mounted system (Neurotargeting Systems) allowing them to move freely in their cage (Fig. 1) as previously described^49^. The cage was positioned on top of the receiver plate that functioned to transfer data from the transmitter to a data exchange matrix (DSI). Following four weeks of postsurgical recovery and 2 days of adaptation to the recording chamber, EEG and EMG signals were recorded via telemetry using Dataquest ART 4.1 software (DSI), analog-to-digital–converted, and stored at 500 Hz on a computer. Continuous EEG/EMG recordings were performed before, during, and after light stimulation of cortical neurons.

### Experimental design

*Experiment 1. Visual light stimulation at 0.6 Hz with 10 ms pulses* Light stimulation with 620-nm light was performed in 2 male and 5 female adult mice at 0.6 Hz with 10 ms pulses for 24 h beginning at ZT3 while sleep–wake recordings continued. EEG/EMG recordings were performed at baseline one day prior to the light stimulation and during the light stimulation.

*Experiment 2. Visual light stimulation at 0.6 Hz and 1.2 Hz with 400 ms pulses* Several days after completing experiment 1, the same mice and additional 4 male adult C57BL/6 J mice were stimulated with 620-nm light pulses at the frequency of 0.6 Hz for either 2 h or 24 h beginning at ZT3. Some of these mice were also stimulated at the frequency of 1.2 Hz in balanced order. Contrary to experiment 1, the duration of light pulses was longer (400 ms). In addition, these mice were subsequently exposed to 730-nm light pulses at the frequency of 0.6 Hz for 2 h.

*Experiment 3. Local optogenetic stimulation of GABAergic neurons in the cerebral cortex at 0.6 Hz with 10 ms light pulses* Two male and 3 female GAD2-cre adult mice were used in this experiment. These mice received AAV viral vector (AAV2/5-Syn-FLEX-rc[ChrimsonR-tdTomato] injection (0.5 μl) into the cerebral cortex. Light stimulation was performed unilaterally to excite cortical interneurons transduced with ChrimsonR using 629-nm LED light source implanted on the skull. Software programmed ATTINY85 microcontroller generated pulses were used to drive the LED light stimulation. The light stimulation of ChrimsonR-transduced cortical cells was done at 0.6-Hz frequency and 10-ms pulse duration for a 24-h period beginning at ZT3 while sleep–wake recordings continued. EEG/EMG recordings were performed at baseline one day prior to the light stimulation and during light stimulation. Immediately after completion of the light stimulation, the mice were perfused with phosphate buffered saline (PBS) and 10% (vol/vol, HT 5011, Sigma) formalin solution for immunohistochemistry.

*Experiment 4. Local optogenetic stimulation of GABAergic neurons in the cerebral cortex at 0.6 Hz with 400 ms light pulses* Several days after completing experiment 3, the same mice were stimulated with 629-nm light at the frequency of 0.6 Hz for 24 h. Contrary to experiment 3, the duration of light pulses was longer (400 ms).

### Sleep data analysis

Data file saved in DSI software was imported into SleepSign software (Kissei Comtec) for further scoring of sleep states in 5-s epochs. Sleep scoring during the 24-h recording of light stimulation was compared to baseline/mock (no LED light stimulation) for both 620-nm/629-nm and 730-nm light source. Vigilance states—including NREM sleep, REM sleep, and waking—were determined in 3-h time blocks as previously described^49^ as one of the following: (1) Wake; active behavior accompanied by desynchronized EEG (low in amplitude), and tonic/phasic motor activity evident in the EMG signal; (2) NREM sleep; more synchronized EEG, higher in amplitude, with particularly notable power in the delta (0.5–4.5 Hz) band, and low motor activity (EMG); and (3) REM sleep; small amplitude EEG, particularly notable power in the theta (4–9 Hz) band, and phasic motor activity (EMG). Compared to wake, EEG power during REM sleep was significantly reduced in delta frequencies (0.5–4.5 Hz) and increased in the range of theta activity (4–9 Hz). The measures included calculating the amount and % time spent in wake, NREM sleep or REM sleep of the 24-h recording during the light stimulation period and baseline/mock period (no light stimulation). As indices of the consolidation of behavioral states, the average duration and number of bouts for each state were calculated.

### EEG data analysis

Power spectral density for each 5-s EEG epoch were computed for each sleep–wake state using a Fast Fourier transform (FFT) after using a Hanning window, yielding power density spectra with a frequency resolution of 0.12207 Hz. Epochs containing artifacts (more than 10xSEM) were excluded from spectral analysis. To account for inter-individual differences in absolute EEG power spectra, power spectra density (PSD) in each frequency bin and for each state was expressed as a percentage of a reference value, calculated across the 24-h period for each individual mouse as the mean total EEG power spectra across all frequency bins (0.5 to 20 Hz) and the behavioral states^50^.Thus, EEG power spectra were normalized by the total power (0.5–20 Hz) for each stage, expressed as a percentage of total power spectra within the 0.5–20 Hz range, and presented in 0.12207 Hz bins in the frequency range of 0.5–20 Hz for either 24-h or 2-h periods. Because responses to visual stimulation did not differ between GAD2-cre mice and C57BL/6 J mice as well as between male and female mice, they were analyzed within the same treatment groups.

Besides SleepSign software (Kissei Comtec), custom codes written in MATLAB 2018b (Natick MA USA) were used in the analyses. Power spectral densities (PSDs) were computed using the multi-taper method (Chronux toolbox; http://chronux.org) as described previously^51^. Additionally, we performed analysis of 1-h long signal segment containing 0.6-Hz square wave and/or 1.2-Hz sine wave stimulus by sliding fast Fourier transforms with a Hanning window using IGOR program (IGOR Pro software, WaveMetrics).

### Immunohistochemistry

Mice were anesthetized, exsanguinated with ice-cold PBS, and perfused transcardially with 10% (vol/vol) formalin (HT5011; Sigma). Brains were post-fixed for 1 day in 10% (vol/vol) formalin followed by incubation in 30% sucrose in PBS for a second day. Coronal slices (40 μm thick) were collected in one of four series and stored in cryoprotectant at 4 °C. In the control slices, primary antibodies were omitted, but were incubated with secondary antibodies only.

To assess the distribution of AAV-transduced neurons, sections were incubated with rabbit anti-red fluorescent protein (RFP) antibody (1:2,000; catalog #600–401-379-RTU, Rockland Immunochemicals) in blocking solution (Donkey serum, 5%; Triton X-100, 1%; PBS, 1x) overnight at room temperature. The next day, sections were incubated in secondary antibody (1:500 biotinylated donkey anti-rabbit IgG; catalog no. 711–005-152; Jackson ImmunoResearch) for 2 h at room temperature and then in an avidin–biotin peroxidase complex (ABC Vectastain Elite kit; catalog no. PK-6100; Vector Labs) for 2 h. tdTomato (RFP) immunoreactivity was visualized with 3,3′-diaminobenzidine tetrahydrochloride (DAB) substrate (Vectastain; catalog no. SK-4100; Vector Labs) in the presence of nickel chloride, which resulted in a gray-black reaction product. Sections were mounted on slides, air dried, dehydrated, cleared in xylene, and coverslipped using Cytoseal 60 (Richard Allen Scientific). Images were acquired using an Olympus BX61 microscope.

Double immunostaining of RFP and cFos were performed on 40 µm-thick brain slices to verify both the AAV transduction and to detect the neuronal activity. The primary antibodies were rabbit anti-RFP (1:2000) (600-401-379-RTU, Rockland Immunochemicals) and rabbit anti-cFos (1:1000) (sc-253, Santa Cruz Biotechnology). The secondary antibody was anti-Rbt-Biotin (1:500) (AP132B, Chemicon International). The ABC (PK-6100, Vector Laboratories Inc., Burlingame, CA) and DAB (SK-4100, Vector Laboratories) staining was performed to visualize the cFos labeled cells (black color in the nuclei). The ABC-AP and Vector Red AP Substrate (AK-5000, Vector Laboratories) was used to visualize the RFP-expressing cells (red color in the cytoplasm).

### Statistical analysis

Comparisons between baseline/mock and light stimulation groups in light stimulation experiments were performed using two-tailed paired t-tests^52^. Statistical analysis used SPSS software (release 11.5), and differences were determined to be significant when *p* < 0.05. Sleep–wake distribution and EEG power spectra were assessed using two-way repeated-measures analysis of variance (rANOVA) as an omnibus test. Tukey’s post-hoc test was then used to determine significant effects. Results are reported as mean ± SEM.

## Supplementary Information

Supplementary Video 1.

Supplementary Information 1.

Supplementary Video 2.

## Data Availability

The raw EEG and EMG recording data are available at https://drive.google.com/drive/folders/1buE3V8nY0SjFHNvTduYF53TfPNetw1GC?usp=sharing. All data generated or analyzed during this study are included in this published article (and its Supplementary Information files). Datasets not included are available from the corresponding author upon reasonable request.
