# Supplementary material for: Low frequency visual stimulation enhances slow wave activity without disrupting the sleep pattern in mice
Source: Sci Rep. 2022 Jul 19;12:12278. doi: 10.1038/s41598-022-16478-8 (PMC9296645; doi:10.1038/s41598-022-16478-8)
Supplement: Supplementary file 2 — Supplementary Information 1. [file 41598_2022_16478_MOESM2_ESM.docx]

**Supplementary Information**

**Examples of programs for producing different patterns of light stimulation.**

(1) Program for generating 10 ms 0.6 Hz flashes using Arduino Uno:

// Blinks an LED connected to pin 2

int blinkPin = 2;

void setup()

{

pinMode(blinkPin, OUTPUT);

}

void loop()

{

digitalWrite(blinkPin, HIGH);

delay(10);

digitalWrite(blinkPin, LOW);

delay(1657);

}

(2) Program for generating 400 ms 0.6 Hz flashes during the period of 24 hours using ATTINY85 microcontroller:

// Blinks an LED connected to pin 0

unsigned long blinks = 0;

void setup()

{

pinMode(0, OUTPUT);

while (blinks <= 51829) {

digitalWrite(0, HIGH);

delay(400);

digitalWrite(0, LOW);

delay(1267);

blinks++;

}

digitalWrite(0, LOW);

while (true) {

// Nothing

}

}

void loop() {}

**Supplementary videos**

Video 1: Visual stimulation with 10-ms 620-nm light pulses at the frequency of 0.6 Hz in a mouse during sleep.

Video 2: Local stimulation of neurons in the cerebral cortex with 400-ms 629-nm light pulses at the frequency of 0.6 Hz in a freely moving mouse.

**Supplementary figures**


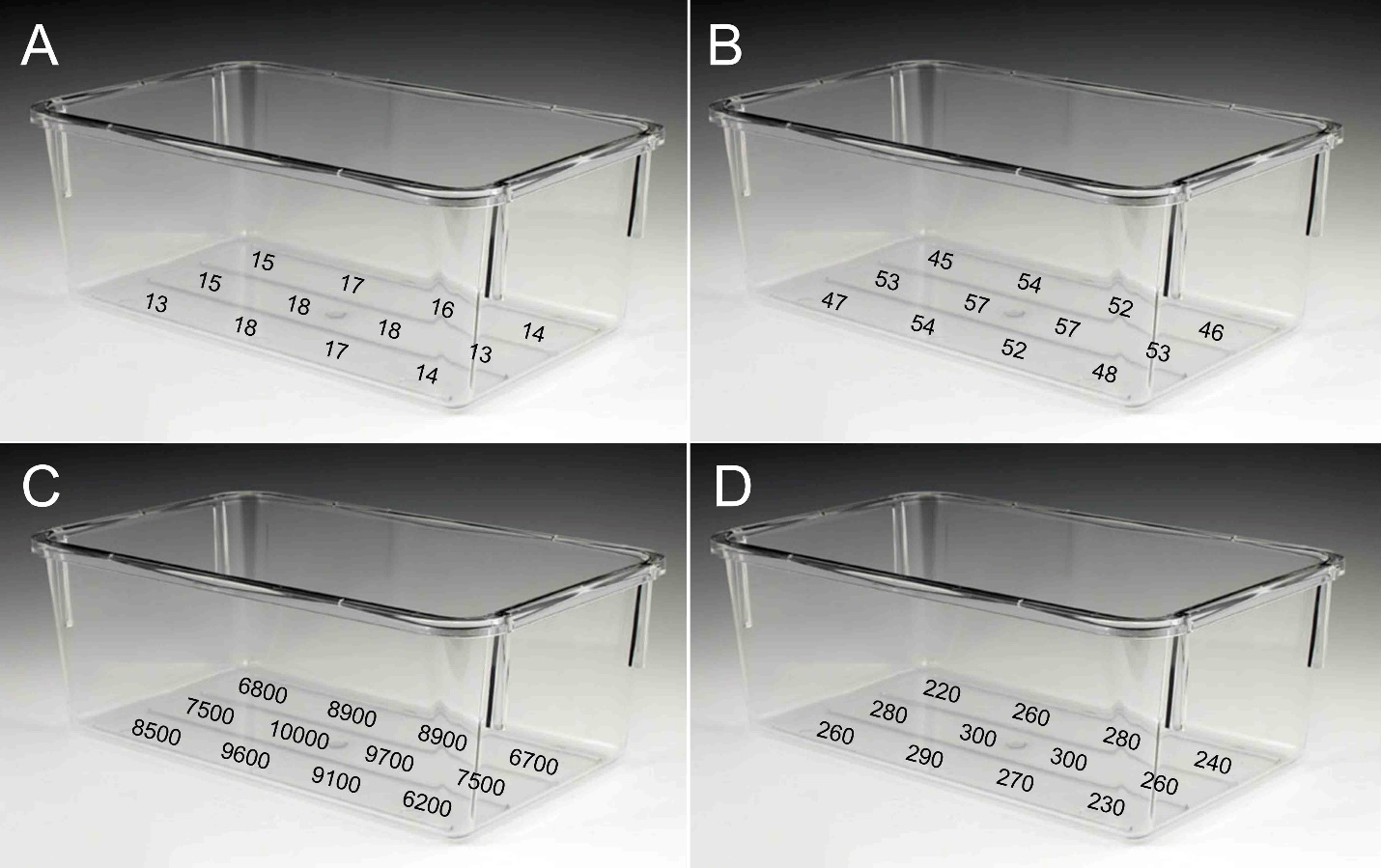


**Supplementary Figure 1.** Light intensity of 620-nm and 730-nm light in different locations of the cage. Measurements were performed by positioning the light sensor in different locations at the bottom of the regular mouse cage. A perforated aluminum plate with attached LED lamps was placed at the top of the cage, and both the LEDs and cage were moved into a dark box. The PM120D meter was used to measure intensity of 620-nm light (A) and 730-nm light (B), and luxmeter was used measure intensity of 620-nm light (C) and 730-nm light (D). The power is presented in µW/mm^2^ (A and B) and in lux (C and D).

**
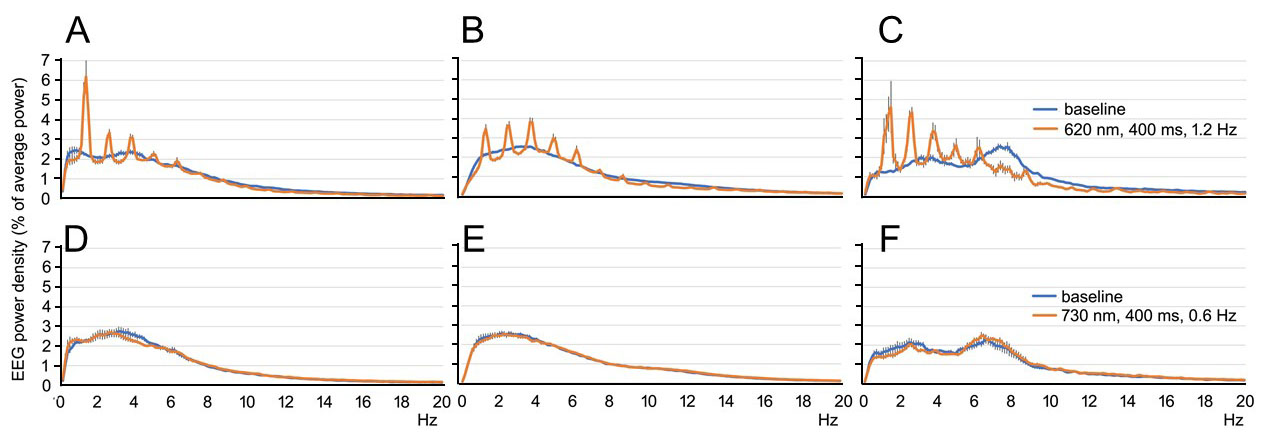
**

**Supplementary Figure 2.** Effect of visual stimulation with 400-ms 620-nm light pulses at 1.2 Hz and 400-ms 730-nm light pulses at 0.6 Hz on EEG power spectrum. A strong response at the stimulation frequency of 1.2 Hz and multiple harmonics were observed in mice exposed to 400-ms 620-nm light pulses at 1.2 Hz during wakefulness (A), NREM sleep (B) and REM sleep (C). No change on the spectrogram was present in response to 400-ms 730-nm light pulses at 0.6 Hz during wakefulness (D), NREM sleep (E) and REM sleep (F).


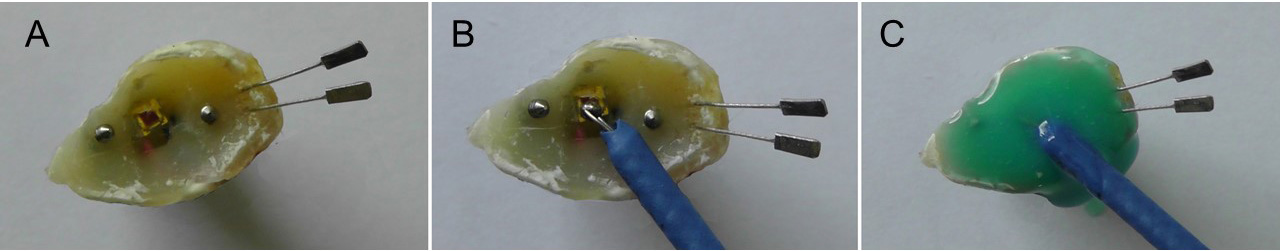


**Supplementary Figure 3.** Temperature measurements under the LED. Five implants containing the 629-nm LED were used in this experiment. In each implant (A), a temperature probe was placed directly under the LED (B) and fixed to the implant with silicone rubber (C). Temperature measurements were performed for 5 min after powering the LED with different flashing patterns. Measurements were repeated in about 5-10 min after the implant cooled down to the room temperature level.


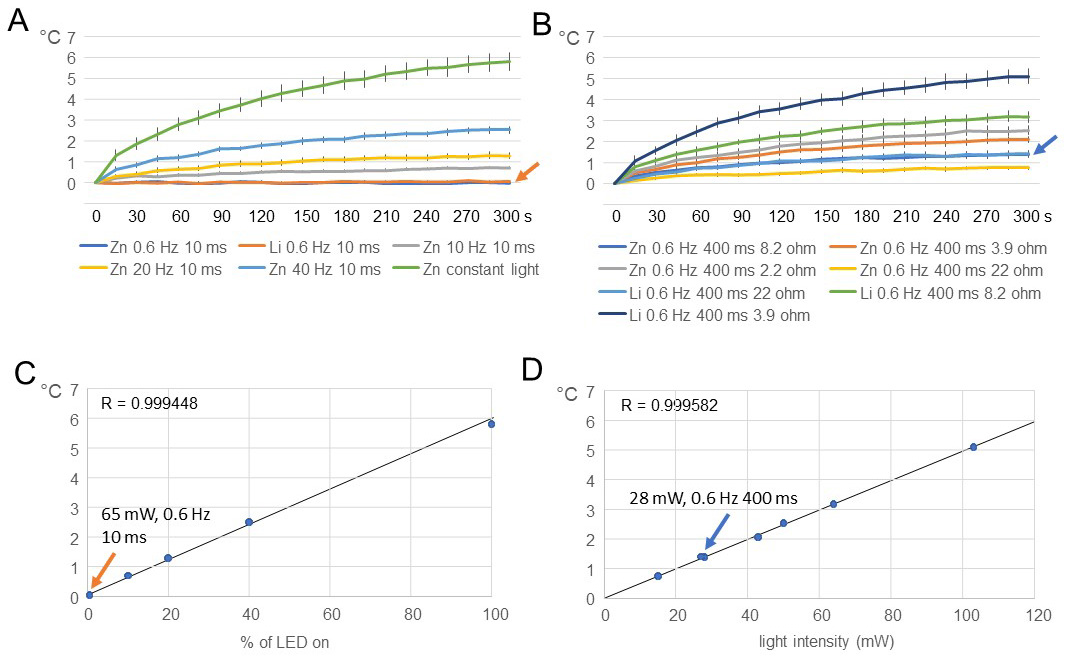


**Supplementary Figure 4.** Effect of LED (629nm) light intensity, duration of light pulses, and frequency of light pulses on temperature. Temperature was measured during the period of 5 min under the LED powered with either 10-ms or 400-ms pulses of current at various frequencies as shown in Supplementary Figure 3. Constant light produced 5.8±0.4°C increase in temperature at the end of the 5-min period, whereas changes in temperature in response to 10-ms light pulses at the low frequency of 0.6 Hz were below the detection limit (A). Temperature increases depended on how much current is supplied to the LED, which was adjusted by using different types of batteries and resistors in the current study (B). Temperature increase was found to be proportional to the time of the LED powered on (C) and light intensity (D). Based on these results, we selected the parameters for LED powering that would be sufficient to stimulate ChrimsonR-containing neurons while not overheating the brain tissue. Orange arrows indicate the parameters used in Experiment 3, whereas blue arrows indicate the parameters used in Experiment 4. Zn, zinc-air battery (675P, ZeniPower). Li, CR2 lithium manganese dioxide battery (EL1CR2BP, Energizer).

**
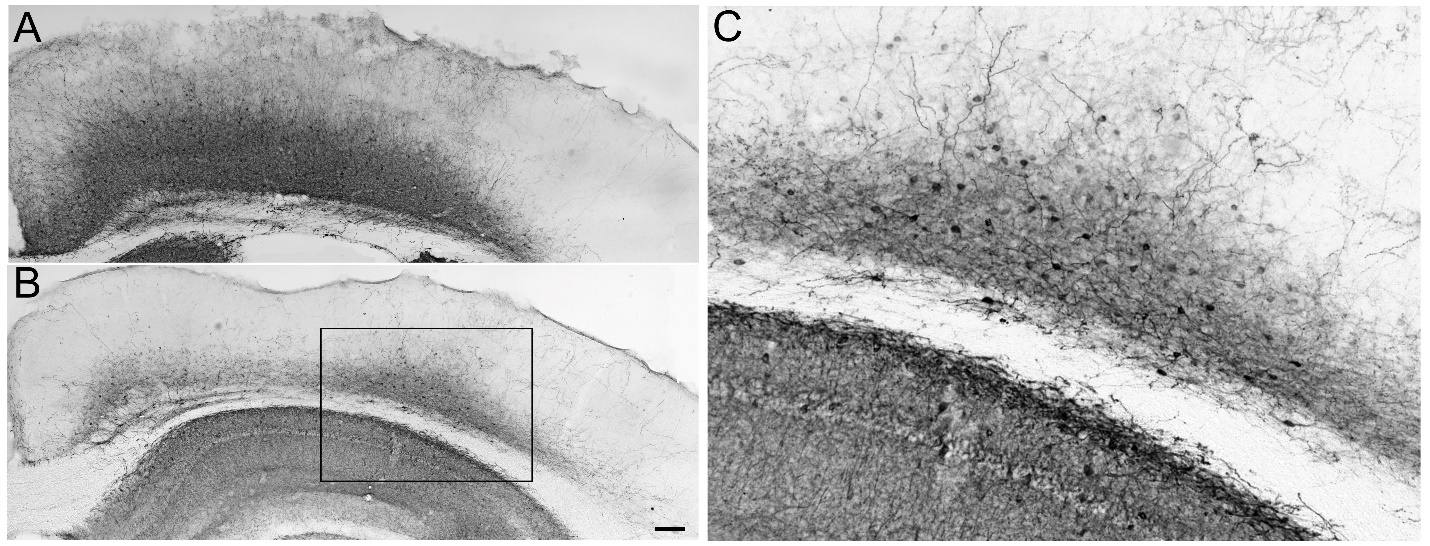
**

**Supplementary Figure 5.** Injection site of pAAV-Syn-FLEX-rc[ChrimsonR-tdTomato] in the cerebral cortex. Location of AAV-expressing cells was identified by the RFP immunostaining. The highest concentration of RFP-positive cells was seen in the deep layers of the cerebral cortex, and some RFP-positive cells were also present in the hippocampus (A and B). The insert (C) shows the rectangle area in (B) at a higher magnification. Scale bar = 200 µm.


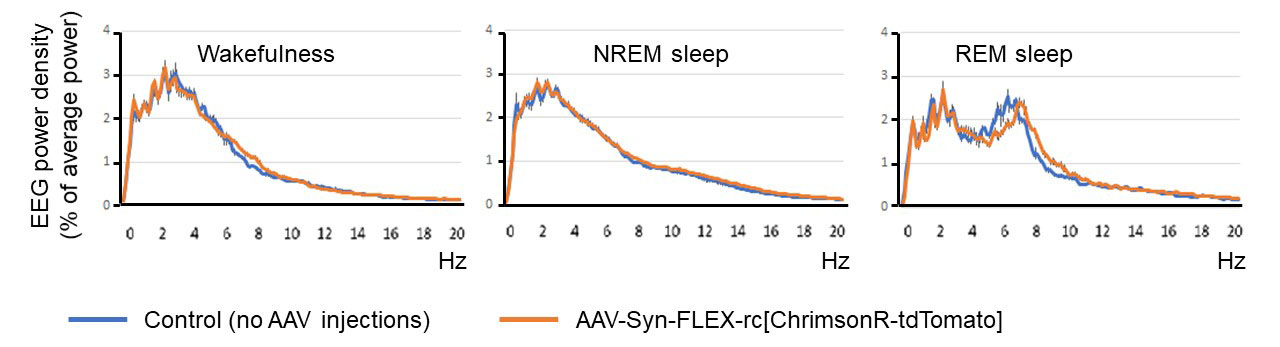


**Supplementary Figure 6.** Occurrence of fundamental and harmonic components on FFT-generated power spectrum in both AAV-injected and control mice. The stimulation frequency peak at 0.6 Hz and harmonics were present not only in mice injected with AAV-Syn-FLEX-rc[ChrimsonR-tdTomato] (n=5 mice, orange line) but also in control mice (n=3 mice, blue line) in which the AAV was not injected into the cerebral cortex.


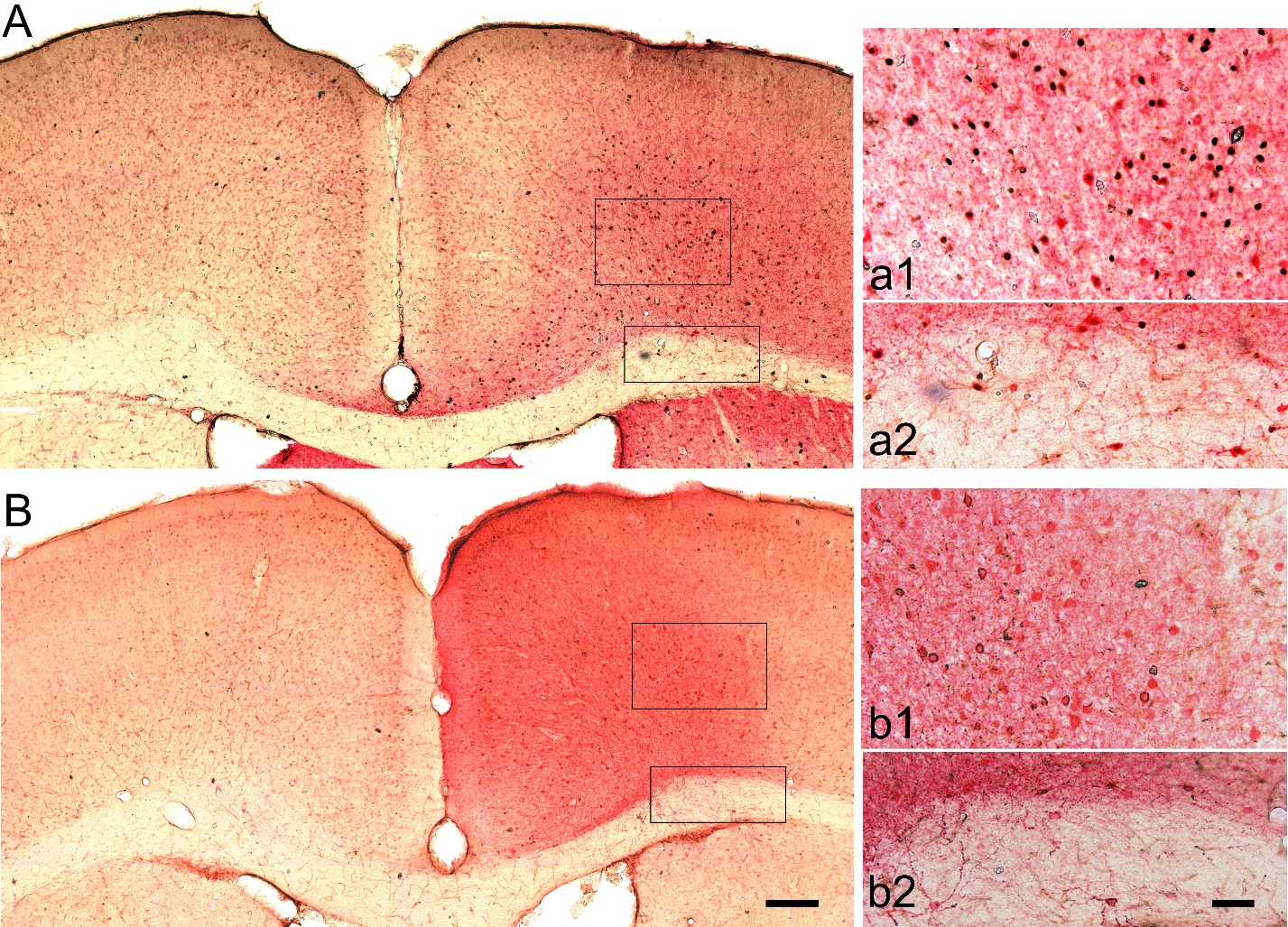


**Supplementary Figure 7.** Representative image of cortical tissue stained for cFos (black) and RFP (red) in mice injected with ChrimsonR-RFP (A) or RFP (B) and exposed to 400-ms 620-nm light pulses at 0.6 Hz for 2-2.5 hours. The image shows that 620-nm light stimulation induced a massive increase in cFos expression at the ChrimsonR-RFP injection site (A), but not the RFP injection site (B). (a1 and a2) A high magnification image of the area shown in A (boxes). (b1 and b2) A high magnification image of the area shown in B (boxes). Scale bars, B: 200 μm (also applies to A); b2: 50 μm (also applies to a1, a2, ans b1).


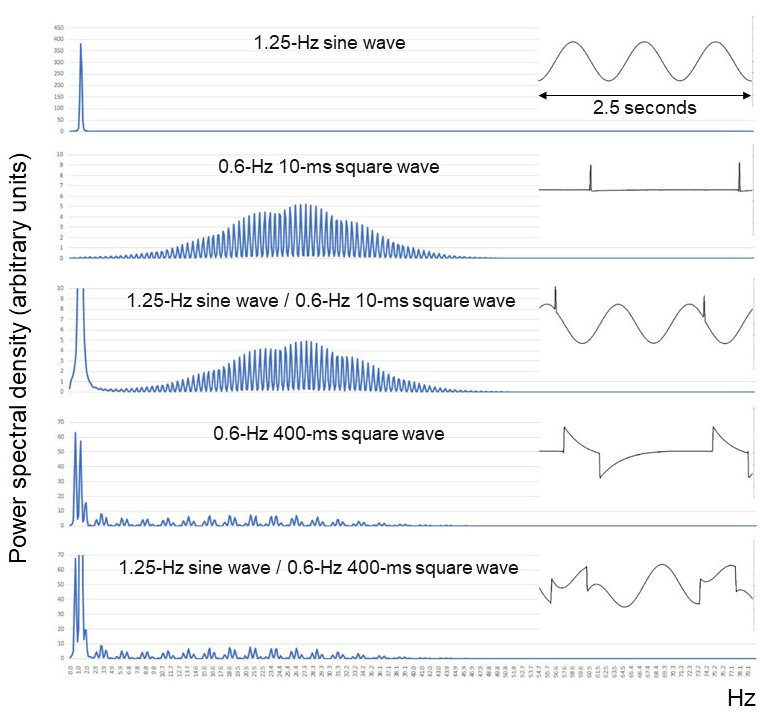


**Supplementary Figure 8.** A fast Fourier transform (FFT) of different waveform data using SleepSign program: 1.25-Hz sine wave / 0.6-Hz square wave. A 2.5-s interval of the recorded waveform is shown in the right upper corner of each FFT plot. FFT of the 1.25-Hz sine wave resulted in a single peak at 1.25 Hz. FFT of the 0.6-Hz square wave produced both the peak at 0.6 Hz and multiple harmonics. Majority of harmonics had a higher amplitude than the peak of the fundamental frequency of 0.6 Hz.


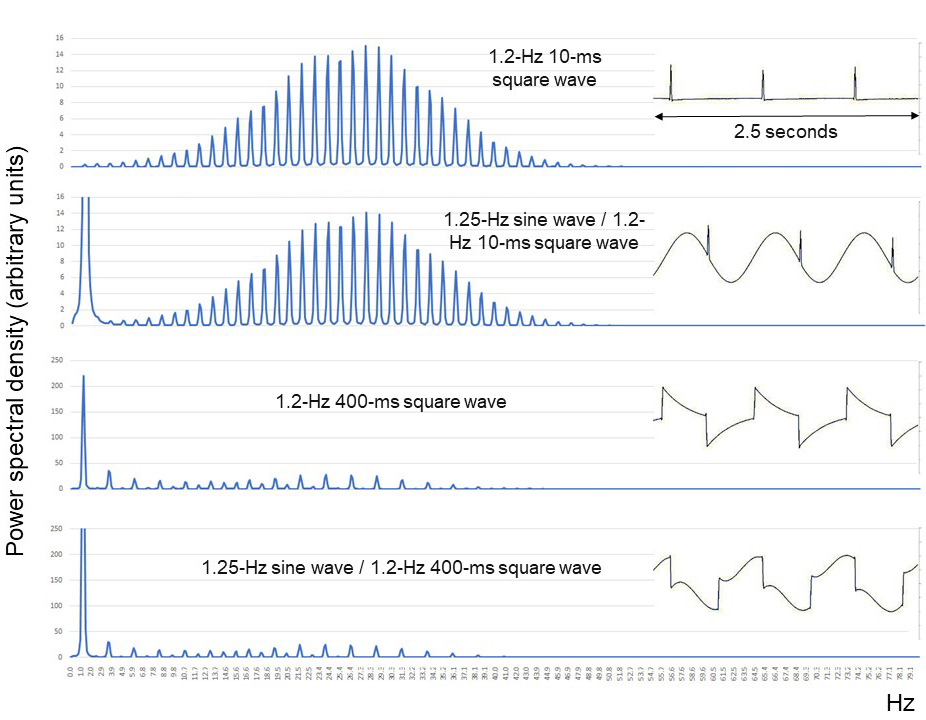


**Supplementary Figure 9.** An FFT of different waveform data using SleepSign program: 1.25-Hz sine wave / 1.2-Hz square wave. Both 10-ms and 400-ms square waves generated harmonics.


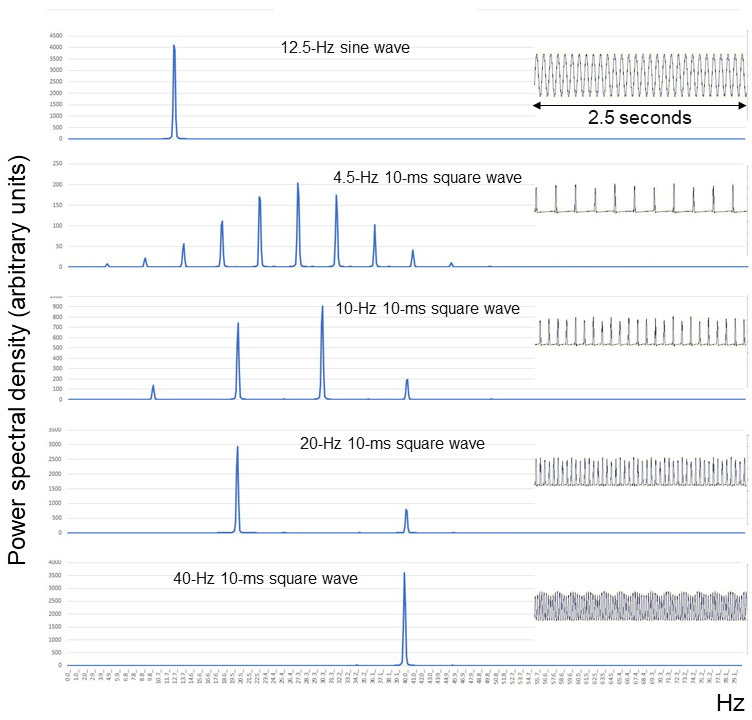


**Supplementary Figure 10.** An FFT of different waveform data using SleepSign program: 12.5-Hz sine wave; 4.5, 10, 20 and 40-Hz square wave. FFT of the 12.5-Hz sine wave resulted in a single peak at 12.5 Hz. FFT of the 4.5, 10, 20 and 40-Hz square wave produced both the peak at fundamental frequency and multiple harmonics.


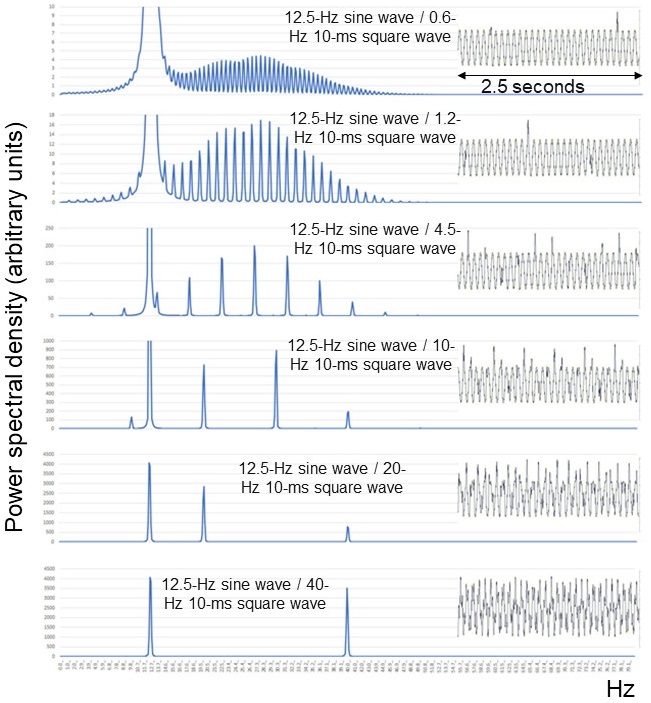


**Supplementary Figure 11.** An FFT of different waveform data using SleepSign program: 12.5-Hz sine wave / 0.6, 1.2, 4.5, 10, 20 and 40-Hz square wave. FFT analysis of the 12.5-Hz sine wave and 10-ms square waves at various frequencies resulted in a single peak at 12.5-Hz peak and multiple harmonics associated with the decomposition of square signals.


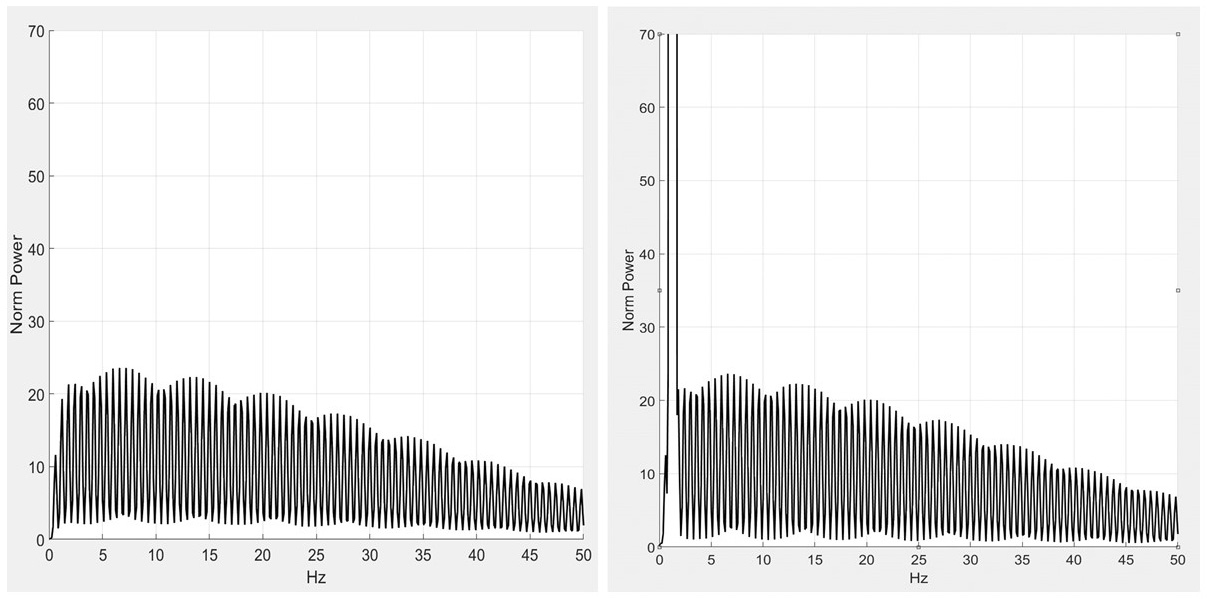


**Supplementary Figure 12.** Multitaper power spectral density analysis: 1.2-Hz sine wave / 0.6-Hz square wave. FFT of the sine wave produced a single peak, whereas FFT of the square wave produced both the peak at fundamental frequency and multiple harmonics.


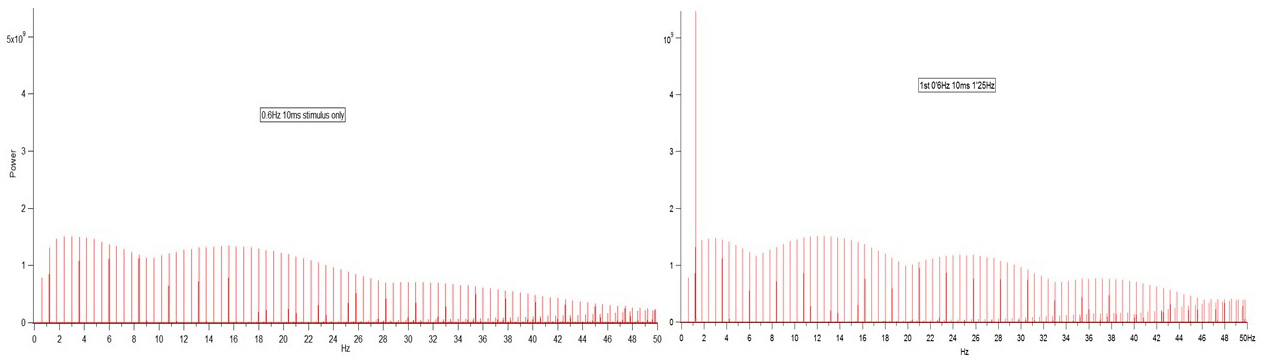


**Supplementary Figure 13.** An FFT with a Hanning window using IGOR program: 1.2-Hz sine wave / 0.6-Hz square wave. FFT of the sine wave produced a single peak, whereas FFT of the square wave produced both the peak at fundamental frequency and multiple harmonics.
